# A comparison of deep versus awake tracheal extubation in adults: a randomized controlled trial

**DOI:** 10.1186/s12871-025-03224-6

**Published:** 2025-07-31

**Authors:** Michael A. Lee, Peter W. Schenke, Matthew R. Faller, Henry R. DeYoung, Ashley R. Adams

**Affiliations:** 1 Naval Medical Center Camp Lejeune, 100 Brewster Blvd Camp Lejeune, Jacksonville, NC 28547 USA; 2 Naval Medical Center Portsmouth, 620 John Paul Jones Circle, VA 23708 Portsmouth, USA

**Keywords:** General endotracheal anesthesia, Awake extubation, Deep extubation, Airway complications, Adult anesthesia, Randomized controlled trials

## Abstract

**Background:**

Awake extubation is deemed a generally safe option for tracheal extubation in low-risk patients, while anesthetized, or“deep” extubation is often considered best suited to seasoned providers due to its perceived hazards. However, inadequate data exists about the relative safety of deep and awake extubations in the adult population.

**Methods:**

Adult patients (*n*=220) with favorable airways undergoing elective surgeries under general tracheal anesthesia were enrolled prospectively. Following a standardized volatile anesthetic regimen, patients were randomized to undergo deep or awake tracheal extubation. The primary outcome was the occurrence of at least one airway or respiratory complication. Secondary outcomes included airway interventions, hemodynamic parameters, severe emergence agitation, and sore throat severity.

**Results:**

Pre-extubation, awake extubations exhibited an increased rate of any airway and respiratory complications (risk ratio [RR] – 5.1; 95% confidence interval [CI] – 2.8-9.5; *p*<.001), attributable to greater incidences of cough (RR – 6.8; 95% CI – 3.2-14.3, *p*>.001) and hypoxemia (RR – 3.6; 95% CI – 1.3-10.6, *p*=.010). After extubation, a significantly decreased rate of one or more complications occurred in the awake extubation group (RR – 0.7; 95% CI – 0.6-1.0; *p*=.028). Awake extubations were associated with fewer incidences of airway obstruction (RR – 0.2; 95% CI – 0.1-0.4, *p*<.001) and apnea (RR – 0.3; 95% CI – 0.1-0.9; *p*=.025), but higher rates of any severity cough (RR – 2.9; 95% CI – 1.6-5.2; *p*<.001). Awake extubations required significantly fewer airway interventions after extubation (RR – 0.2; 95% CI – 0.1-0.6; *p*<.001). No serious adverse events occurred.

**Conclusions:**

Deep and awake extubations produce distinct airway and respiratory complication profiles, without either being conclusively safer. The risks posed by each technique during emergence and after extubation should be considered by anesthesia providers when formulating an extubation strategy.

**Trial registration:**

This study was retrospectively registered at clinicaltrials.gov (NCT05361850) on April 23, 2022.

## Background

During the provision of general anesthesia, the safe removal of an endotracheal tube (ETT) is as critical as its proper placement. Complications such as obstruction, aspiration, laryngospasm, coughing, hypoxemia, apnea, and bronchospasm can occur to varying degrees; of major complications associated with anesthesia, nearly 30% were during extubation or emergence [1]. The overall complication rate during extubation may in fact be higher than that of intubation [2]. Despite these risks, there is a comparative lack of studies honing routine extubation techniques for adults. Many fundamental details like optimum positioning, method of secretion removal, application of positive pressure, and fraction of inspired oxygen have yet to be elucidated [3]. The ideal anesthetic plane at the time of ETT removal remains a contestable matter – whether to extubate ‘awake’ following the return of airway reflexes and spontaneous movement, or ‘deep’ with unresponsiveness to noxious stimuli.

Some perceived benefits of adult deep extubation (DE) are hemodynamic stability, minimization of coughing/bucking, and attenuation of bronchospasm, while commonly cited reasons to perform an awake extubation (AE) include risk mitigation of airway obstruction, laryngospasm, and aspiration [4]. Investigations comparing the two techniques have shown that DE may reduce intra-abdominal pressure following hernia surgery [5] and emergence agitation following nasal surgery [6]. Unfortunately, prospective randomized clinical trials (RCTs) have not thoroughly studied the effect of extubation method on airway or respiratory complications in an adult population. Research efforts in pediatric anesthesia have generally concluded that there are no differences in overall airway and respiratory complications, and that both extubation methods are acceptable [7–10]. With regards to adult patients, a prospective survey conducted in the 1990 s observed that airway and respiratory complications were more likely with DE than AE, although the possibility of reporting bias limits definitive conclusions [2]. More recently, an observational study involving 300 adult patients undergoing DE demonstrated a low (13%) complication rate but lacked an AE comparator group [11]. The only pertinent RCT for adult patients enrolled sixty subjects with noted demographic differences between the three groups [12]. Exclusion criteria, sample size, and medications in use at that time also moderates the clinical applicability of this study to modern anesthetic care.

The decision to perform DE in lieu of AE for an adult is thus largely determined by the anesthesiologist’s professional judgment and experience, rather than data guiding best practices. With DE and AE being regularly performed in adult surgical patients [4], opinions of the techniques and their relative safety require more substantiation. We therefore designed this prospective RCT to directly compare the airway and respiratory complication rates of awake and deep endotracheal extubations in adult surgical patients with low-risk airways in a conventional perioperative setting.

## Methods

Naval Medical Center Portsmouth’s Institutional Review Board approved this study, which was retrospectively registered at clinicaltrials.gov (NCT05361850) on April 23, 2022, and reported using CONSORT guidelines [13]. This parallel study at two Defense Health Agency medical centers randomly assigned participants 1:1 to AE or DE. Adults scheduled for elective surgery indicating general tracheal anesthesia from August 28, 2020, to August 9, 2023, with favorable airway examinations (Mallampati class 1–2), normal mouth opening, adequate cervical range-of-motion, preoperative fasting adherence, and American Society of Anesthesiologist (ASA) physical status 1–3 were contacted for recruitment by phone prior to day-of-surgery. To avoid undue coercion that may result from the hierarchical nature of the military, recruiting investigators referred to themselves by their professional medical title, not military rank. The voluntary nature of participation was emphasized during recruitment and was explicitly delineated on the written informed consent, which was obtained day-of-surgery.

Patients were ineligible if undergoing intrathoracic procedures, airway surgery, prone positioning, surgery with anticipated duration over six hours, receiving a protocolized intraoperative Enhanced Recovery After Surgery anesthetic, had history of difficult mask ventilation or intubation, pregnancy, chronic obstructive pulmonary disease, pulmonary hypertension, interstitial lung disease, active respiratory infection, uncontrolled asthma, or uncontrolled gastro-esophageal reflux disease (GERD).

Before study initiation, study identification numbers (IDs) were reordered via computer-generated randomization. For a 1:1 randomization scheme resulting in similar sample sizes, 110 IDs were allotted to AE and 110 to DE. Sealed envelopes labelled with IDs contained the assigned extubation method. Consenting patients were sequentially assigned an ID. Allocation occurred 20 min from anticipated end-of-surgery, when a sealed opaque envelope labelled with the ID was opened by the anesthetist to reveal the assigned extubation method.

### Study procedures

The standardized intravenous induction regimen consisted of midazolam 0.02–0.04 mg/kg, lidocaine 1-1.5 mg/kg, fentanyl 1–2 mcg/kg, propofol 1–2 mg/kg and rocuronium 0.6–1.2 mg/kg. Additional medications were allowed at the discretion of the anesthetist. After induction, ease of mask ventilation was assessed. Use of an oropharyngeal or nasopharyngeal airway was permissible. Patients were intubated utilizing direct laryngoscopy. Trainees were permitted one attempt at laryngoscopy. Maintenance of anesthesia was accomplished with sevoflurane titrated to an age-adjusted minimum alveolar concentration greater than 0.5, hydromorphone 2–6 mcg/kg as needed for pain, rocuronium boluses as needed, and additional medications at the anesthetist’s discretion.

Subjects were disenrolled if mask ventilation required a two-hand technique or was otherwise difficult, three or more attempts were made, or a technique other than direct laryngoscopy was used. The anesthetist could withdraw the patient from the study if they believed the assigned extubation method would result in an unacceptable risk of harm. In that event, data would be excluded from final analysis.

When approaching end-of-surgery, ondansetron was given unless contraindicated. Antagonism of neuromuscular blockade was accomplished with sugammadex 2–4 mg/kg as determined by train-of-four monitoring. Spontaneous respirations with adequate minute ventilation were established for all patients, as judged by the anesthetist. No significant changes to the anesthetic regimen, such as initiation of a remifentanil or dexmedetomidine infusion, were allowed.

For patients undergoing AE, a soft bite block of rolled gauze was placed to prevent biting on the ETT. Deep oropharyngeal suction was performed with a rigid Yankauer suction catheter. The sevoflurane vaporizer was switched to the “off” position and fraction of inspired oxygen was changed to 100%. Upon spontaneous eye opening or purposeful movement, the ETT cuff was deflated, and the patient was extubated. During removal of the ETT, positive airway pressure of 10 cmH_2_0 or greater was achieved by compressing the reservoir bag.

For patients undergoing DE, the age-adjusted minimum alveolar concentration was increased to one or greater by titration of sevoflurane and fraction of inspired oxygen was changed to 100%. Deep oropharyngeal suction was performed with a rigid Yankauer suction catheter. Pre-emptive placement of an oropharyngeal or nasopharyngeal airway was at the anesthetist’s discretion. The ETT cuff was deflated to assess non-responsiveness, as defined by continued regular respirations without gross motor response. If responsive, the anesthetist deepened the plane of anesthesia by increasing the inspired sevoflurane concentration. Once non-responsive to suction and cuff deflation, the patient was extubated. During removal of the ETT, positive airway pressure of 10 cmH_2_0 or greater was achieved by compressing the reservoir bag.

Following all extubations, the anesthetist confirmed airway patency and adequate ventilation and oxygenation. No specific instructions were given as to airway management, with the anesthetist performing appropriate and clinically indicated interventions as needed. Data collection for both arms continued in the operating room (OR) until the patient emerged from anesthesia, as defined by spontaneous eye opening with a reliably patent airway and no ongoing complications. After emergence, the patient was transferred to the Post Anesthesia Care Unit (PACU). All data was collected in the OR by an unblinded study investigator, as the anesthetic plane at extubation is difficult to conceal.

During transport, patients received supplemental oxygen at 6 L/minute via simple face mask in the supine position with head-of-bed elevated. Ventilation was monitored by the anesthetist. Turnover from anesthesia to PACU was conducted per hospital protocol. PACU personnel were informed of the patient’s study enrollment but not extubation method. Upon meeting phase one criteria, the patient was discharged from the PACU, with no planned follow-up.

### Variables and measurements

Subjects’ age, biological sex, body mass index (BMI), ASA physical status, vital signs, and comorbidities (specifically, GERD, obstructive sleep apnea (OSA), active smoking within the last four weeks, asthma, and obesity) were recorded. BMI was calculated from patient height and day-of-surgery weight. The procedure type, start- and end-of-surgery times, out-of-room time, and PACU arrival and discharge times were documented. End-of-surgery was defined by drape breakdown and final application of surgical dressings.

Vitals signs, complications, and interventions were recorded at baseline, within the ten minutes prior to extubation (pre-extubation), at extubation, immediately following extubation while the patient remained in the OR (post-extubation) and in the PACU. Heart rate, blood pressures and S_p_O_2_ were measured at all time periods. End tidal carbon dioxide and ventilatory frequency were recorded during pre-extubation and at time-of-extubation, while F_i_O_2_ was collected only pre-extubation.

Complications included cough, obstruction, hypoxemia, apnea, bronchospasm, and laryngospasm. Obstruction was defined as narrowing or occlusion of the airway by supraglottic anatomical structures resulting in impaired ventilation. Cough severity was determined by the Modified Minogue Scale (MMS) [14], with severe cough considered a score of three or four. Hypoxemia was classified as mild (91–94%), moderate (86–90%), severe (80–85%) or critical (79% or less) as measured by any pulse oximetry reading with an appropriate plethysmographic waveform. Bronchospasm was defined as deleterious spasmodic bronchial smooth muscle contraction, and laryngospasm as partial or complete airway structure resulting from maladaptive reflex closure of the vocal cords. Apnea was defined as cessation of respiratory effort for ten seconds or longer. Sedation was measured using the Richmond Agitation-Sedation Scale (RASS) [15], with severe agitation being determined by a score of three or greater. Serious adverse events included aspiration, persistent hypoxemia, re-intubation, hemodynamic instability requiring continued vasopressor infusion, unplanned intensive care unit admission or death.

Interventions included chin lifts, positive airway pressure, two-hand jaw thrust, oral or nasal airway use, and medications. Any continuous positive airway pressure, supported ventilation, or manual ventilation was considered positive airway pressure.

Prior to extubation, all vital signs collected were transcribed from the intraoperative record onto the data collection tool in two-minute intervals for the ten minutes preceding extubation, once actual extubation time was known. Complications, including emesis, were recorded if they occurred at any time within ten minutes before extubation. Intraoperative medications not otherwise specified, hypoxemia, and use of nitrous oxide were recorded if present on either on the intraoperative record or data collection tool.

Post-extubation vital signs, oxygen source, complications, and interventions were recorded in two-minute intervals from extubation until PACU transfer.

Vital signs, complications, and airway interventions were documented upon arrival, in three five-minute intervals and a final fifteen-minute interval, to capture the first 30 min of PACU admission. Immediately before PACU discharge, the patient graded their sore throat on a 0–10 numeric pain rating scale. Serious adverse events were recorded over the whole study duration.

### Statistical analysis

The primary outcome was overall complication rate, defined as the occurrence of at least one airway or respiratory complication, independent of the type or severity of complication. In a similarly designed pediatric study [7], the overall complication rates were 74% for AE patients and 56% for DE patients. To have a 95% chance of detecting a difference in overall complications (assuming one exists) between adult patients undergoing AE versus DE, with p-value of < 0.05 considered significant, 95 participants per arm were necessary. To account for withdrawal or dropout, 220 participants were recruited.

Analysis was conducted with SPSS 28.0 (IBM Corp, Armock NY). Numerical data were reported in means and standard deviations or medians with first and third quartiles and compared between DE and AE arms using t-tests with or without variance correction, or the Wilcoxon Rank Sum as needed. Categorical data were reported in counts, frequencies, risk ratios (RRs) and 95% confidence intervals (95%CI), and compared between arms using Χ^2^ or Fisher’s Exact as appropriate.

To account for the varying amount of time before patients were transferred to PACU, analyses of complications after extubation were reported with hazard ratios (HRs) and 95% confidence intervals and analyzed with log-rank tests and Kaplan-Meier curves. Data was censored if the patient was transferred to the PACU without experiencing a complication. Cox regression analyses were used to determine if patient or procedural characteristics were confounding variables in the survival analyses.

Vital statistics were analyzed with repeated measures ANOVA, using the Greenhouse-Geisser correction if sphericity could not be assumed. P-values less than 0.05 were considered statistically significant and all applicable tests were two-tailed. Missing data were treated as missing and not imputed.

##  Results

**Fig. 1:**
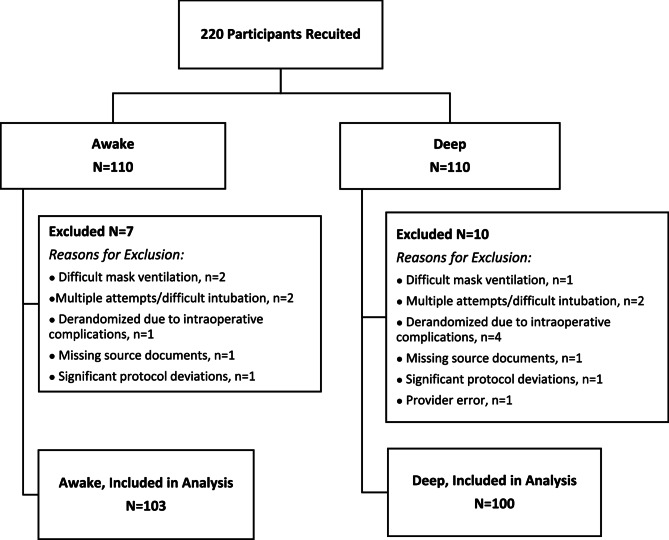
Flowchart of participant enrolment into both the AE and DE arms

Of 419 patients screened for eligibility, 220 patients were enrolled. Seventeen were excluded for difficult ventilation or intubation, intraoperative complications, or issues of protocol (Fig. 1). The analysis includes 103 AE participants and 100 DE participants. Patients were in their mid-thirties, split between male and female, and were ASA II (138/203, 68.0%), with obesity (63/203, 31.0%) and GERD (42/203, 20.7%) being the most common comorbidities (Table 1). Participants had similar pre-operative vitals, comparable anesthetic medications, and were admitted predominately under orthopedics (88/203, 43.3%, Table 2).

**Table 1:** Demographics of patients in AE and DE arms

Variable		Awake N=103	Deep N=100
Age (years)		35.6 (11.1)	34.3 (10.4)
Gender	*Female*	48 (46.6)	49 (49.0)
	*Male*	55 (53.4)	51 (51.0)
Weight (kg)		83.3 (17.3)	81.6 (14.5)
Height (cm)		172.2 (10.3)	171.5 (9.2)
BMI (kg/m^2^)		28.0 (4.9)	27.6 (4.1)
ASA	*1 – Healthy*	24 (23.3)	32 (32.0)
	*2 – Mild Systemic*	72 (69.9)	66 (66.0)
	*3 – Severe Systemic*	7 (6.8)	2 (2.0)
Comorbidities	*GERD*	18 (17.5)	24 (24.0)
	*Obstructive Sleep Apnea*	10 (9.7)	14 (14.0)
	*Active Smoker*	22 (21.4)	16 (16.0)
	*Asthma*	9 (8.7)	3 (3.0)
	*Obesity*	33 (32.0)	30 (30.0)
	*Other Comorbidities*	10 (9.7)	11 (11.0)

Data reported in N (%) and mean (SD)

During the pre-extubation period, defined by the ten minutes prior to extubation, the overall complication rate was significantly higher for AE patients than DE patients (RR: 5.1; 95%CI: 2.8–9.5, *p* <.001, Table 3). This difference remained significant when a single cough (MMS = 2) was removed from the overall pre-extubation complication rate (AE 39.8% vs. DE 5.0%; RR: 8.0; 95%CI: 3.3–19.3, *p* <.001). AE patients had higher rates of cough (RR: 6.8; 95%CI: 3.2–14.3; *p* <.001), severe cough (RR: 16.5; 95%CI: 4.1–66.9; *p* <.001), and any hypoxemia (RR: 3.6; 95%CI: 1.3–10.6; *p* =.010). Additionally, 5.8% of AE patients experienced severe agitation prior to ETT removal, compared to 0% in DE patients (*p* =.029).

**Table 2:** Intubation and procedural characteristics of patients in the AE and DE arms

Variable	Awake N=103	Deep N=100	*P*-value
Pre-operative vitals*			
Heart rate	74.7 (12.8)	76.4 (12.3)	
Oxygen saturation	98.3 (1.5)	98.3 (1.3)	
Systolic blood pressure	124.5 (13.6)	124.7 (12.7)	
Diastolic blood pressure	79.7 (8.2)	80.4 (9.9)	
Mean arterial pressure	94.6 (9.3)	95.5 (8.7)	
Induction medications			
Midazolam	103 (100.0)	98 (98.0)	
Fentanyl	102 (99.0)	97 (97.0)	
Lidocaine	99 (96.1)	97 (97.0)	
Propofol	103 (100.0)	100 (100.0)	
Rocuronium	99 (96.1)	97 (97.0)	
Dexamethasone	98 (95.1)	94 (94.0)	
Intubation and ventilation			
Placement of oral airway	13 (12.6)	19 (19.0)	
Placement of nasal airway	0 (0.0)	1 (1.0)	
Intubated by trainee (1^st^ Attempt Only)	40 (38.8)	44 (44.0)	
Intubated by staff (1^st^ or 2^nd^ Attempt)	62 (60.2)	56 (56.0)	
Maintenance medications			
Sevoflurane	103 (100.0)	100 (100.0)	
Hydromorphone	75 (72.8)	66 (66.0)	
Total hydromorphone	1.10 (0.60)	1.03 (1.01)	
Rocuronium	99 (96.1)	97 (97.0)	
Total rocuronium	73.0 (35.3)	64.6 (26.8)	
Ketorolac	42 (40.8)	39 (39.0)	
Nitrousꝉ	13 (13.0)	8 (8.5)	
Other maintenance medication	77 (74.8)	78 (78.0)	
Emergence medications			
Ondansetron	101 (98.1)	100 (100.0)	
Sugammadex	96 (93.2)	92 (92.0)	
Other emergence medication	21 (20.4)	15 (15.0)	
Surgical service			
* General surgery*	24 (23.3)	23 (23.0)	
* Orthopedic*	47 (45.6)	41 (41.0)	
* Gynecologic*	18 (17.5)	22 (22.0)	
* Plastics*	9 (8.7)	7 (7.0)	
* Urologic*	5 (4.9)	6 (6.0)	
* ENT*	0 (0.0)	1 (1.0)	
Surgical times (mins)			
Procedure length	124.5 (66.2)	111.1 (58.7)	.128
End of surgery to extubation	3.05 (6.2)	-3.87 (6.6)	<.001
Extubation to out of room	5.20 (2.8)	12.8 (5.2)	<.001
End of surgery to out of room	8.25 (5.8)	8.93 (6.5)	.433

Data reported in N (%) and mean (SD)

*N(A) = 103, N(B)=99

ꝉN(A)=100, N(B)=94. ENT = Otorhinolaryngology

During the post-extubation period in the operating room, the overall complication rate was significantly decreased in the AE arm (RR: 0.7; 95%CI: 0.6-1.0; *p* =.028, Table 3). This difference remained significant when a single cough (MMS = 2) was removed from the overall post-extubation complication rate (AE 32.9% vs. DE 67.1%, RR: 0.5; 95%CI: 0.3–0.7, *p* <.001). AE participants experienced less obstruction (RR: 0.2; 95%CI: 0.1–0.4; *p* <.001) and apnea (RR: 0.3; 95%CI: 0.1–0.9, *p* =.025), but higher incidences of cough (RR: 2.9; 95%CI: 1.6–5.2; *p* <.001).

**Fig. 2:**
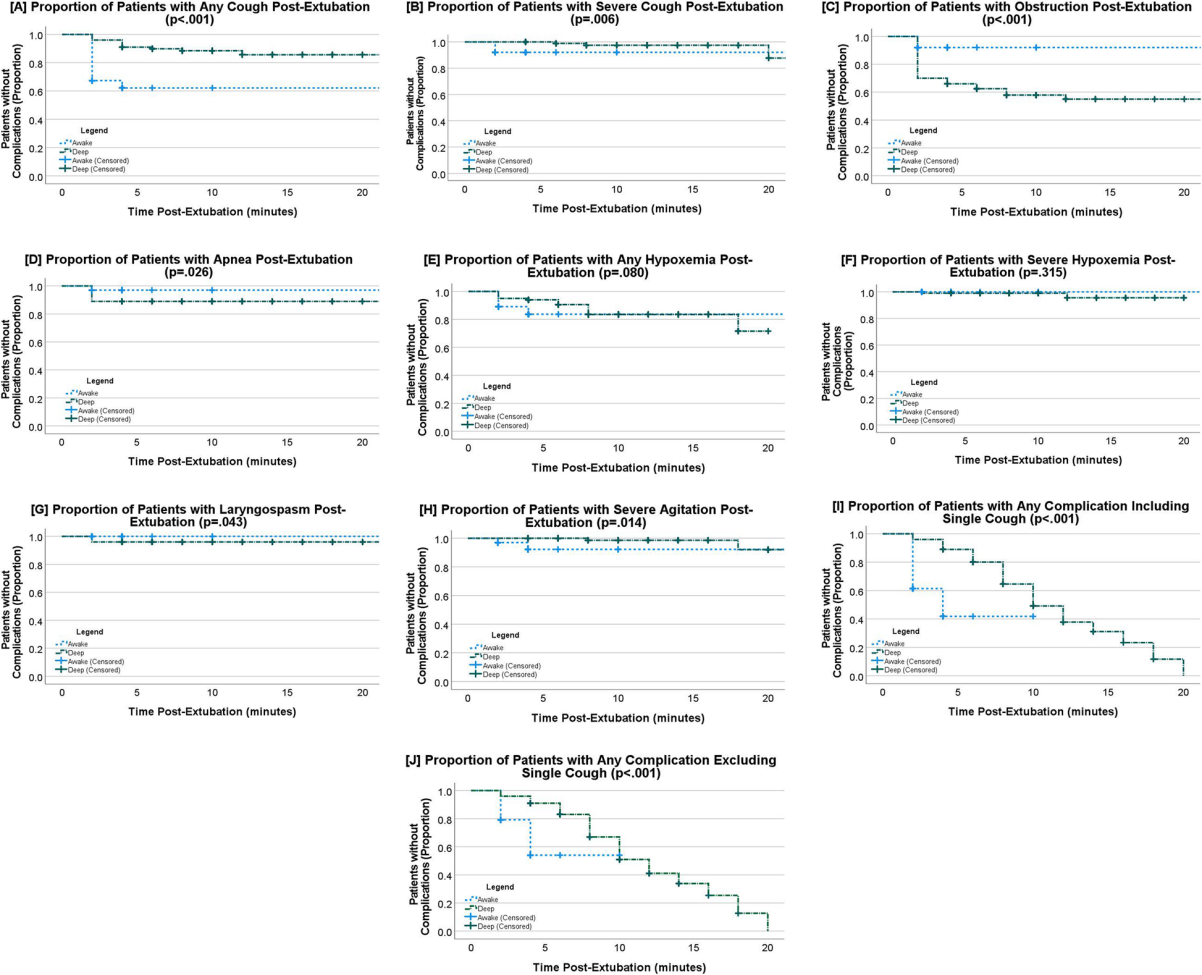
Survival curves of complications after extubation to PACU Transfer; analysis with Log-Rank test. **A** Any Cough (*p* <.001) **B** Severe Cough, Modified Minogue Score of 3 or 4 (*p* =.006) **C** Obstruction (*p* <.001) **D** Apnea (*p* =.026) **E** Any Hypoxemia, < 94% (*p* =.080) **F** Severe Hypoxemia, < 85% (*p* =.315) **G** Laryngospasm (*p* =.043) **H** Severe Agitation (*p* =.014). **I** Overall Complications (*p* <.001). **J** Overall Complications, excluding Modified Minogue Score of 2 (*p* <.001). There were no cases of Bronchospasm

Survival analyses found similar results, with the odds of post-extubation complications being significantly less likely in AE patients as compared to DE patients for any cough (HR: 0.20; 95%CI: 0.09 − 0.04; *p* <.001; Fig. 2A), severe cough (HR: 0.08; 95%CI: 0.01–0.60; *p* =.006, Fig. 2B), and severe agitation (HR: 0.06; 95%CI: 0.00-0.80; *p* =.014, Fig. 2H), and significantly greater in DE patients for obstruction (HR: 4.09; 95%CI: 1.89–8.83; *p* <.001, Fig. 2C) and apnea (HR: 3.70; 95%CI: 1.03–13.3; *p* =.026, Fig. 2D). Overall complications post-extubation were significantly less likely in the AE arm (HR: 0.20; 95%CI: 0.12–0.36, *p* <.001, Fig. 2I), even when a single cough (MMS = 2) was removed from the analysis (HR:0.25; 95%CI: 0.13–0.48, *p* <.001, Fig. 2H). Analysis of patient and procedural characteristics, such as sex or comorbidities, found no significant confounding variables.

While laryngospasm episodes only occurred in DE patients immediately following extubation (DE 4.0% vs. AE 0%), this was not statistically significant (*p* =.059). In all four laryngospasm episodes involving DE, the patients were initially reactive to oropharyngeal suction or tracheal cuff deflation. Anesthesia was deepened in accordance with study criteria, yet laryngospasm still occurred.

**Table 3:** Complications and interventions for patients in the AE and DE arms

	Awake N=103	Deep N=100	Relative Risk of Awake	*P*-value
Pre-extubation complications				
Any cough	49 (47.6)	7 (7.0)	6.8 [3.2-14.3]	<.001
Severe cough (MMS 3 or 4)	34 (33.0)	2 (2.0)	16.5 [4.1-66.9]	<.001
Any hypoxemia	15 (14.6)	4 (4.0)	3.6 [1.3-10.6]	.010
Severe hypoxemia (<85%)	2 (1.9)	0 (0)	N/A	.498
Bronchospasm	0 (0)	0 (0)	N/A	N/A
Severe agitation	6 (5.8)	0 (0)	N/A	.029
Any respiratory complication*	53 (51.5)	10 (10.0)	5.1 [2.8-9.5]	<.001
Any respiratory complication* (excluding MMS=2)	41 (39.8)	5 (5.0)	8.0 [3.3-19.3]	<.001
Post-extubation complications				
Any cough	35 (34.7)	12 (12.0)	2.9 [1.6-5.2]	<.001
Severe cough (MMS 3 or 4)	8 (7.9)	3 (3.0)	2.6 [0.7-9.7]	.125
Obstruction	8 (7.9)	42 (42.0)	0.2 [0.1-0.4]	<.001
Apnea	3 (3.0)	11 (11.0)	0.3 [0.1-0.9]	.025
Any hypoxemia	13 (12.9)	15 (15.0)	0.9 [0.4-1.7]	.663
Severe hypoxemia (<85%)	0 (0)	2 (2.0)	N/A	.246
Laryngospasm	0 (0)	4 (4.0)	N/A	.059
Bronchospasm	0 (0)	0 (0)	N/A	N/A
Severe agitationꝉ	5 (5.0)	2 (2.0)	2.5 [0.5-12.6]	.445
Any respiratory complication*	46 (45.5)	61 (61.0)	0.7 [0.6-1.0]	.028
Any respiratory complication* (excluding MMS=2)	28 (32.9)	57 (67.1)	0.5 [0.3-0.7]	<.001
Post-extubation interventions				
Chin lift	34 (33.7)	88 (88.0)	0.4 [0.3-0.5]	<.001
Positive pressure	32 (31.7)	69 (69.0)	0.5 [0.3-0.6]	<.001
Jaw thrust	10 (9.9)	30 (30.0)	0.3 [0.2-0.6]	<.001
Oral nasal air	3 (3.0)	62 (62.0)	0.05 [0.01-0.15]	<.001
Medications	2 (2.0)	2 (2.0)	1.0 [0.1-6.9]	1.000
Post-anesthesia care unit (PACU) complications				
Any cough	12 (11.7)	7 (7.0)	1.7 [0.7-4.1]	.255
Severe cough (MMS 3 or 4)	2 (1.9)	1 (1.0)	1.9 [0.2-21.1]	1.00
Obstruction	3 (2.9)	3 (3.0)	1.0 [0.2-4.7]	1.00
Apnea	2 (1.9)	0 (0)	N/A	.498
Any hypoxemia	25 (24.3)	20 (20.0)	1.2 [0.7-2.0]	.464
Severe hypoxemia (<85%)	1 (1.0)	3 (3.0)	0.3 [0.0-3.1]	.364
Laryngospasm	1 (1.0)	1 (1.0)	1.0 [0.1-15.3]	1.00
Bronchospasm	0 (0)	0 (0)	N/A	N/A
Severe agitation	1 (1.0)	0 (0)	N/A	1.00
Any respiratory complication*	36 (35.0)	25 (25.0)	1.3 [0.9-1.8]	.122
PACU interventions				
Chin lift	7 (6.8)	5 (5.0)	1.4 [0.4-4.1]	.587
Positive pressure	1 (1.0)	1 (1.0)	1.0 [0.1-15.3]	1.00
Jaw thrust	2 (1.9)	3 (3.0)	0.6 [0.1-3.8]	.680
Oral nasal air	5 (4.9)	20 (20.0)	0.2 [0.1-0.6]	.001
Medications	10 (9.7)	12 (12.0)	0.2 [0.1-0.6]	.600
PACU length of stay (mins)	55.0 [35.0-76.0]	52.5 [37.3-76.8]		.833
Sore throat (Scale of 0-10)	0.0 [0.0-2.0]	0.0 [0.0-1.0]		.242

Data reported in N (%), Mean [Q1-Q3], or Relative Risk [95% Confidence Interval]

*Does not include Severe Agitation

ꝉN(Awake) = 100, N(Deep)=100. MMS = Modified Minogue Scale

Post-extubation, AE patients received fewer chin lifts (RR: 0.4; 95%CI: 0.3–0.5; *p* <.001, Table 3), positive pressure ventilation (RR: 0.5; 95%CI: 0.3–0.6; *p* <.001), jaw thrusts (RR: 0.3; 95%CI: 0.2–0.6, *p* <.001), and use of oral/nasal airway adjuncts (RR: 0.05; 95%CI: 0.01–0.15 *p* <.001). There was no significant difference in post-extubation medication administration.

Analysis of vital statistics did not measure if vitals changed over time, but if changes were different between groups. AE and DE arms had significantly different changes in heart rate (*p* <.001, Table 3), systolic blood pressure (< 0.001), diastolic blood pressure (*p* <.001), and mean arterial pressure (*p* <.001). Specifically, DE patients had more consistent measurements and fewer increases, while AE patients increased as they approached extubation. There was a significant difference in F_i_O_2_ change between arms (*p* =.001, Table 3), where the F_i_O_2_ was similar ten minutes prior to extubation but increased more rapidly over time in the AE arm. S_p_O_2_, respiratory rate, and end-tidal carbon dioxide changes were similar.

At the time of extubation, more AE patients experienced hypertension (MAP ≥ 110 mmHg, 19.8% vs. 0%, *p* <.001) and tachycardia (HR ≥ 100 beats per minute, RR: 6.9; 95%CI: 2.3–20.8; *p* <.001), while fewer patients experienced hypotension (MAP ≤ 65 mmHg, RR: 0.65; 95%CI: 0.48–0.87; *p* =.022). Bradycardia occurred more frequently in DE patients (HR ≤ 60 beats per minute, RR: 0.67; 95%CI: 0.46–0.96; *p* =.086) but not significantly so.

Procedure lengths and end-of-surgery to out-of-room times were similar (Table 2). AE occurred 3.05 [SD 6.15] minutes after end-of-surgery and DE occurred 3.87 [SD 6.56] minutes prior (*p* <.001); however, DE had significantly longer extubation to out-of-room time (12.8 [SD 5.21] minutes vs. 5.20 [SD 2.80] minutes, *p* <.001).

Overall rates of PACU complications were not significantly higher in AE (RR: 1.3; 95%CI: 0.9–1.8, *p* =.122), though significantly fewer AE patients had oral/nasal airways on PACU arrival or during phase one recovery (RR: 0.2; 95%CI: 0.1–0.6; *p* =.001, Table 3). The changes of PACU vital statistics were not significantly different. There was a significant difference in how the RASS changed between AE and DE patients (*p* =.001), where DE patients were admitted at significantly lower average RASS (−1.7 vs. −1.1, *p* =.003), but improved more rapidly, until the average in each arm was similar after 30 min (Table 4).

No patient experienced a serious adverse event.

**Table 4:** Vital statistics before and after extubation in the AE and DE arms

Vital		Minutes to Extubation								*P*-value
		-10	-8	-6	-4	-2	0	2	4	
Systolic blood pressure (mmHg)	*Awake* *N=21*	109.3 (12.8)	110.6 (13.5)	112 (13.0)	115.4 (15.5)	118.9 (16.3)	123.9 (19.6)	128.8 (21.8)	133.1 (17.3)	<.001
	*Deep* *N=71*	104.6 (12.4)	104.7 (12.6)	104.9 (12.6)	103.5 (13.6)	103.8 (12.7)	104.6 (13.8)	110.8 (15.7)	111.4 (13.5)	
Diastolic blood pressure (mmHg)	*Awake* N=23*	61.3 (11.5)	62.2 (11.9)	62.7 (12.1)	64.4 (13.6)	68.5 (16.4)	74.7 (18.6)	74.2 (15.3)	68.7 (16.1)	<.001
	*Deep* *N=71*	57.4 (12.4)	56.6 (12.7)	55.9 (12.1)	56.1 (12.2)	55.4 (12.4)	55.9 (12.6)	60.5 (14.6)	60.2 (12.0)	
Mean arterial pressure (mmHg)	*Awake* *N=24*	80.1 (10.3)	81.7 (12.0)	82.1 (12.1)	83.9 (13.2)	88.0 (15.1)	94.8 (17.4)	96.1 (17.2)	97.6 (15.7)	<.001
	*Deep* *N=72*	76.5 (11.5)	76 (11.9)	75.7 (10.6)	75.4 (11.8)	75.1 (11.5)	75.7 (11.1)	80.4 (13.2)	80.1 (11.5)	
Heart rate (beats/min)	*Awake* *N=35*	71.8 (11.9)	72.8 (10.6)	71.8 (10.5)	73.1 (11.6)	78.0 (15.1)	88.7 (18.4)	92.4 (14.7)	87.7 (15.7)	<.001
	*Deep* *N=98*	75.3 (11.8)	74.6 (11.6)	74.1 (11.1)	74.3 (10.9)	74.0 (13.5)	75.7 (12.5)	78.5 (12.8)	78.1 (12.9)	
Oxygen saturation (%)	*Awake* *N=35*	98.6 (1.5)	98.5 (1.8)	98.6 (1.6)	98.7 (1.6)	98.8 (2.0)	99.0 (1.8)	98.6 (2.0)	97.5 (3.2)	.124
	*Deep* *N=97*	98.4 (1.4)	98.5 (1.4)	98.5 (1.4)	98.5 (1.6)	98.5 (1.6)	98.5 (1.5)	98.4 (2.5)	98.4 (2.2)	
Respiratory rate (breaths/min)	*Awake* *N=101*	16.0 (14.1)	14.5 (6.2)	14.5 (4.9)	14.7 (4.6)	15.4 (4.9)	16.7 (6.0)	-	-	.102
	*Deep* *N=95*	13.8 (5.1)	13.3 (5.7)	14.1 (6.2)	15.3 (5.8)	15.7 (5.4)	14.9 (5.3)	-	-	
End-tidal carbon dioxide (mmHg)	*Awake* *N=100*	44.1 (7.2)	44.8 (6.1)	45.5 (5.6)	46.0 (5.2)	45.9 (5.8)	43.7 (7.5)	-	-	.059
	*Deep* *N=95*	44.5 (8.4)	44.5 (9.1)	46.5 (7.5)	46.0 (9.6)	47.8 (8.7)	46.7 (8.3)	-	-	
Fraction of inspired oxygen (%)	*Awake* *N=102*	68.1 (23.2)	74.6 (23.0)	81.0 (20.3)	86.3 (17.7)	93.4 (8.7)	-	-	-	.001
	*Deep* *N=98*	66.8 (19.4)	68.9 (19.4)	72.4 (18.4)	76.7 (17.5)	82.0 (13.8)	-	-	-	

Data reported in mean (SD)

*N represents the count of patients with recorded measures at all time points

## Discussion

During the pre-extubation emergence phase, AE exhibited an increased overall complication rate due to greater rates of cough and hypoxemia. Immediately following extubation, DE presented a greater risk of overall complications owing to more obstruction and apnea, despite AE still correlating with a higher rate of cough. After excluding mild cough as a complication, these differences in complication rates remained statistically significant. Each method had a low overall incidence of severe hypoxemia in the OR, with two patients in each arm desaturating to below 85%. There were no differences in complications during the PACU phase.

Our results correspond to similar studies, and we substantiated DE being associated with more overall complications in the immediate post-extubation period, while indicating a pre-extubation benefit of DE with regards to coughing and hypoxemia. The only other RCT comparing AE and DE airway and respiratory complications in adults found more cough in AE patients and obstruction in DE patients, though the incidences were higher at 90% and 85%, respectively [12]. The continuous period of data collection, from end-of-surgery to PACU discharge, may have contributed to these rates, as well as medications used. A large prospective survey observed a higher incidence of post-extubation airway and respiratory complications in DE patients (17.8%) as compared to AE patients (10.1%) [2]. While our respective rates were higher at 61% and 45.5%, we substantiated this general finding that DE is associated with more overall complications than AE in the immediate post-extubation period. A single-arm observational study of adult DE patients noted an overall complication rate of 13%, considerably less than our 61% rate [11], likely due to stricter definitions of cough and hypoxemia and the exclusion of obstruction as a complication. Our complication rates tracked with that of pediatric literature, with a meta-analysis concluding that AE presented a greater risk of cough while DE posed a greater risk for airway obstruction [16]. 

We noted four laryngospasm episodes immediately following extubation and two on arrival to the PACU, similar to previously reported rates of 1.7% and 0.8% [2]. Notably, all four laryngospasms that occurred immediately following extubation were in DE patients. These subjects were initially reactive to oropharyngeal suction and/or ETT cuff deflation. Anesthesia was then deepened to meet study criteria for DE, and yet these patients laryngospasmed anyways, one to the point of developing critical hypoxemia that required administration of succinylcholine. We therefore caution anesthesia providers that a patient who responds to these provocative maneuvers may continue to be at risk for laryngospasm, even after deepening of the anesthetic plane.

DE patients received significantly more corrective interventions than AE patients. Anesthetists placed airway adjuncts more frequently for DE, although we did not stipulate their routine use. Oropharyngeal airways were not required for DE, as they are not essential to maintain airway patency after DE [17], nor does published evidence comparing DE to a native airway versus DE with an oral airway already in place exist. Utilizing simple airway maneuvers alone conforms to expert guidelines for low-risk DE [18]. 

DE has been proposed as a method to attenuate tachycardia and hypertension during the peri-extubation period [19], but no substantial data establishes this advantage in adults. Our study confirmed Qiu and colleagues’ findings on peri-extubation hypertension [20], with similar proportions of AE patients exhibiting hypertension at time of extubation, but no DE patients. In addition, our AE group had a significantly greater incidence of tachycardia at extubation. However, with increasing evidence detailing the risks of intraoperative hypotension [21, 22], the benefit of reduced tachycardia and hypertension from DE may be offset by the prevalence of hypotension. We noted a propensity for more hypotension in the DE arm at time of extubation, unlike Suo et al. [6]

Aspects of our protocol development warrant further explanation. In powering our study, we selected a pediatric trial that closely resembled our intended study design [7]. This prospective RCT compared DE and AE in high-risk pediatric patients. While not clinically identical, we contend that the similar definition of the primary outcome and inclusion of elevated-risk adult patients made this study most appropriate for power analysis. Our complication rates were similar to this study, reinforcing this decision. We avoided strategies known to facilitate a tranquil emergence, which may have impacted our complication rates, because administering pharmacologic adjuncts unequally between groups might have skewed results [23–27]. Their omission here is not a reflection on their utility, and we advocate these interventions when anesthetists perceive a clinical benefit. Due to our criterion for PACU transfer, AE patients had a shorter duration of post-extubation data collection in the OR, making OR comparisons challenging. Protocol designs, such as keeping patients in the OR for a pre-determined length of time, were considered but rejected for their significant departure from usual OR workflow.

On average, DE patients were extubated before end-of-surgery, while AE patients were extubated after end-of-surgery. Extubation timing was at provider discretion and not protocolized. No data supports or refutes the safety of either extubation method prior to end-of-surgery. Anecdotally, we recognized no issue with DE during the final minutes of surgery, when stimulation was typically limited to superficial skin closure and application of dressings. The increased time required for DE to emerge and meet OR discharge criteria offset any time gained by early DE. Therefore, we observed no difference in extubation method for end-of-surgery to out-of-room time. With similar PACU stay times in both groups (AE 55 min and DE 52.5 min, *p* =.833), we cannot infer any cost savings or improved OR time metrics. Transferring DE patients to the PACU prior to emergence may have reduced total anesthesia time, however. Published data on desaturation rates in the PACU following DE of adults [28] and our data on complications during DE emergence may guide clinician decision-making on the prudence of doing so.

Our study had several strengths. Prospective randomization allowed for similar baseline characteristics. Including patients with GERD, OSA, active smoking, asthma, and obesity made our findings more generalizable than related studies. We devised a standardized anesthetic regimen for this study, ensuring groups received comparable medications. Extubation methods were nearly identical to sequences detailed by the Difficult Airway Society. Withdrawals resulting from significant protocol deviations, difficult initial airway management, or missing source documents were within the expected range. Minor protocol deviations were distributed equally between groups. Randomization occurred near end-of-surgery, limiting opportunities for anesthetists to introduce bias.

A major weakness of our study is observer bias, as the blinding of investigators to the extubation method is unfeasible when evaluating respiratory and airway complications. Video capture and review of the extubation by a blinded assessor was considered but deemed unworkable due to facility limitations. Therefore, an in-person investigator was utilized for data collection, and attempts were made to mitigate bias whenever possible. Complications were precisely defined before study initiation to reduce disagreement. Our investigators had no financial or professional motivations to favor one technique over the other. The primary investigator or an associate investigator was present for data collection at all extubations included in data analysis. The study investigators were responsible for identifying and recording the presence of complications, although clarification could be sought from the clinical anesthesia provider. Save for the protocolized steps of extubation, the clinically responsible anesthesia providers were not coached to perform any specific airway intervention, nor were they encouraged to avoid any particular complications. Nonetheless, pre-existing assumptions [4] may have unconsciously influenced investigators to perceive the ‘expected’ outcomes of DE and AE. However, each complication of interest was still observed in both groups despite potential implicit bias, except bronchospasm and aspiration, which did not occur in our study.

The subjective nature of the primary outcome is another major limitation. Clinicians do not always agree on the diagnosis of a particular complication. Laryngospasm, bronchospasm, and supraglottic airway obstruction may not be readily distinguishable, especially if the presentation is transient or rapidly corrected by vigilant anesthesia providers. These problems may also exist simultaneously. Which complications to include in our analysis is another contestable matter. Is it appropriate to define cough, a usually protective physiologic response, as a complication? Is obstruction indeed a complication if fully resolved by only a gentle chin lift? Likewise, is apnea a problem if oxygenation if easily maintained by mask ventilation until the patient resumes spontaneous respiratory effort? It seems less controversial to label bronchospasm or laryngospasm as complications, but do anesthesia providers routinely document their occurrence if no significant treatment is required, or if there is no consequent hypoxemia? Hypoxemia as a complication is also debatable based on duration, oxygen saturation nadir, and reversibility. These questions also touch on another flaw in evaluating airway and respiratory complication rates, namely that complications are not created equally. We stratified two complication rates by considering the incidence of severe cough and severe hypoxemia in addition to overall rates. Future studies would benefit from the use of additional grading scales, such as degree of laryngospasm or extent of obstruction. Clinicians would then be better equipped to tailor the extubation method and associated risks they deem specifically relevant to their patients. However, we do argue that cough, obstruction, apnea, hypoxemia, laryngospasm, and bronchospasm are all broadly undesirable in the peri-extubation period. Our position is supported by the breadth of anesthetic literature addressing these untoward events, even if they are not considered outright complications by some anesthetists.

## Conclusion

**Fig. 3:**
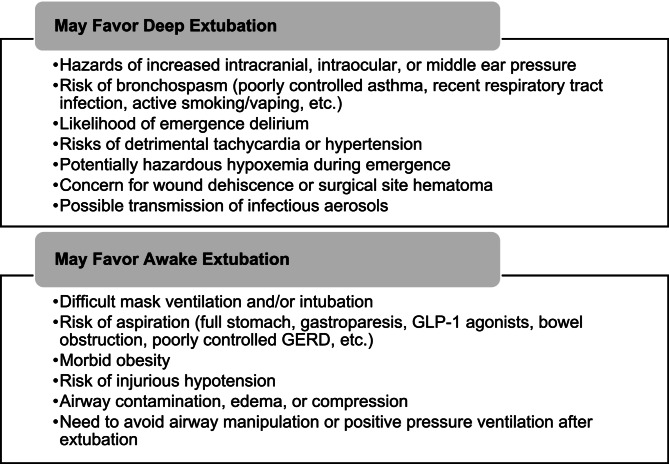
Hypothesized relative indications for extubation method in adults

In adult patients with favorable airways undergoing elective surgeries of the extremities, abdomen, and pelvic region, both AE and DE are suitable approaches to ETT removal. Each technique has advantages in the context of airway and respiratory complications. AE safeguards airway patency and ventilatory drive, resulting in less obstruction, apneic periods, and need for airway interventions following extubation. DE minimizes airway reactivity, with reduced cough and hypoxemia during pre-extubation emergence. We offer a summary of clinical factors that theoretically rationalize either an AE or DE (Fig. 3) but must emphasize that inadequate data support these recommendations [3–6, 19]. Additional studies are necessary to determine the effect of extubation method on these select co-morbidities or surgical procedures. Pending further research into clinical outcomes, we trust the responsible anesthetist’s judgment to decide the preferred extubation method for their patient’s unique situation.

## Data Availability

The datasets used and/or analyzed during the current study are available from the corresponding author on reasonable request.

## Abbreviations

95%CI: 95% confidence interval
AE: Awake extubation
ASA: American Society of Anesthesiology
BMI: Body mass index
CONSORT: Consolidated standards of reporting trials
DE: Deep extubation
ETT: Endotracheal tube
GERD: Gastro-esophageal reflux disease
HR: Hazard ratios
ID: Identification number
OR: Operating room
OSA: Obstructive sleep apnea
PACU: Post-anesthesia care unit
RASS: Richmond agitation-sedation scale
RCT: Randomized controlled trial
RR: Risk ratio
